# The role of change facilitators in the implementation of alcohol SBI with public health nurses

**DOI:** 10.1186/1940-0640-10-S2-O17

**Published:** 2015-09-24

**Authors:** Sherilyn Faulkner, Diane K King, Rebecca Porter, Bridget Hanson

**Affiliations:** 1 Center for Behavioral Health Research and Services, University of Alaska Anchorage, Anchorage, USA

## Background

The University of Alaska Anchorage partnered with the State of Alaska, Section of Public Health Nursing (PHN) to pilot routine alcohol screening and brief intervention (aSBI) in three of their 22 public health centers (PHCs) prior to system-wide dissemination. During this two-year project funded by the Centers for Disease Control and Prevention, the university research team facilitated aSBI implementation through collaborative planning with PHN leadership, providing training for nurses to conduct aSBI, and maintaining contact with PHN staff. Nurses at the pilot sites participated in regular phone calls with members of the research team acting as change facilitators. A Plan-Do-Study-Act approach was used to refine implementation protocols throughout the pilot.

## Materials and methods

A semi-structured protocol was used by change facilitators to identify challenges, successes, and fidelity issues. Each PHC participated in 12-16 calls over 14 months. Content from calls was iteratively discussed by the research team and key issues were presented to PHN's aSBI implementation planning team in order to continuously refine protocols during pilot implementation. Thematic analysis of contact logs revealed key themes.

## Results

Emergent themes included challenges with documentation procedures, on-going training needs, and adoption successes. Feedback from calls resulted in the refinement of PHN's aSBI policies and procedures, supported booster training for nurses to improve brief intervention skills, and were used to develop local aSBI resources for nurses and clients. Throughout the project, nurses expressed increased acceptance of conducting aSBI as a routine part of client visits.

## Discussion

Change facilitation calls provided a structured mechanism to involve nurses in piloting and improving aSBI implementation procedures, and to foster buy-in necessary for the adoption of practice change.

## Conclusions

Change facilitation served as an important method for improving fidelity and feasibility of aSBI within the PHN health system.

